# Transfection of *Babesia bovis* by Double Selection with WR99210 and Blasticidin-S and Its Application for Functional Analysis of Thioredoxin Peroxidase-1

**DOI:** 10.1371/journal.pone.0125993

**Published:** 2015-05-11

**Authors:** Masahito Asada, Kazuhide Yahata, Hassan Hakimi, Naoaki Yokoyama, Ikuo Igarashi, Osamu Kaneko, Carlos E. Suarez, Shin-ichiro Kawazu

**Affiliations:** 1 Department of Protozoology, Institute of Tropical Medicine (NEKKEN), Nagasaki University, Sakamoto, Nagasaki, Japan; 2 National Research Center for Protozoan Diseases, Obihiro University of Agriculture and Veterinary Medicine, Inada, Obihiro, Japan; 3 Program in Vector-Borne Diseases, Department of Veterinary Microbiology and Pathology, Washington State University, Pullman, Washington, United States of America; 4 Animal Disease Research Unit, Agricultural Research Service, United States Department of Agriculture, Pullman, Washington, United States of America; Federal University of São Paulo, BRAZIL

## Abstract

Genetic manipulation is an essential technique to analyze gene function; however, limited methods are available for *Babesia bovis*, a causative pathogen of the globally important cattle disease, bovine babesiosis. To date, two stable transfection systems have been developed for *B*. *bovis*, using selectable markers blasticidin-S deaminase (*bsd*) or human dihydrofolate reductase (*hdhfr*). In this work, we combine these two selectable markers in a sequential transfection system. Specifically, a parent transgenic *B*. *bovis* line which episomally expresses green fluorescent protein (GFP) and human dihydrofolate reductase (hDHFR), was transfected with a plasmid encoding a fusion protein consisting of red fluorescent protein (RFP) and blasticidin-S deaminase (BSD). Selection with WR99210 and blasticidin-S resulted in the emergence of parasites double positive for GFP and RFP. We then applied this method to complement gene function in a parasite line in which thioredoxin peroxidase-1 (*Bbtpx-1*) gene was knocked out using hDHFR as a selectable marker. A plasmid was constructed harboring both RFP-BSD and *Bbtpx-1* expression cassettes, and transfected into a *Bbtpx-1* knockout (KO) parasite. Transfectants were independently obtained by two transfection methods, episomal transfection and genome integration. Complementation of *Bbtpx-1* resulted in full recovery of resistance to nitrosative stress, via the nitric oxide donor sodium nitroprusside, which was impaired in the *Bbtpx-1* KO parasites. In conclusion, we developed a sequential transfection method in *B*. *bovis* and subsequently applied this technique in a gene complementation study. This method will enable broader genetic manipulation of *Babesia* toward enhancing our understanding of the biology of this parasite.

## Introduction

*Babesia bovis* is an intraerythrocytic apicomplexan parasite, which is transmitted by ticks and causes the disease bovine babesiosis in cattle. This disease has a significant global economic and social burden [1–3], and new control strategies are needed. The complete genome sequence of *B*. *bovis* was reported, but the function of more than 50% of the genes remains unassigned [4, 5]. A major obstacle impeding studies of gene function in *Babesia* parasites is the paucity of gene manipulation methodologies [6]. Application of transfection technology and reverse genetics on protozoan parasites has had a major impact on defining gene functions and identifying potential targets for intervention [7]. For example, in apicomplexan parasites such as *Plasmodium* spp. and *Toxoplasma gondii*, several genetic manipulation tools have been developed which are widely used to study gene functions, as well as applied to develop genetically defined attenuated vaccines [8].

The initial work for developing transfection methods of *B*. *bovis* began in the last decade by developing a transient transfection method using the *rap-1* or *ef-1α* promoter and the terminator of the *rap-1* genes of *B*. *bovis* controlling expression of luciferase as a reporter [9–11]. Subsequently, stable transfection of a plasmid construct expressing a fusion protein consisting of green fluorescent protein (GFP) and blasticidin-S deaminase (BSD) and targeted integration of the construct into the parasite genome was achieved following selection with blasticidin-S [12, 13]. It was further demonstrated that the integrated *gfp-bsd* gene can be expressed and remain stable upon inoculation of transgenic parasites into calves [14]. Alternative transfection constructs designed for targeted epitope expression and using a bidirectional promoter have also been developed [15]. Applying another selection marker human dihydrofolate reductase (hDHFR), conferring resistance to WR99210, successful disruption was achieved of the thioredoxin peroxiredoxidase-1 (*Bbtpx-1*) gene locus by a homologous recombination approach [16]. This study described a *B*. *bovis Bbtpx-1* knock out (KO) clonal line which is able to grow in *in vitro* culture despite lacking expression of the *Bbtpx-1* gene.

In the present study, we combine blasticidin-S/*bsd* and WR99210/*hdhfr* selection systems within a sequential transfection methodology for *B*. *bovis*. We show that the developed sequential transfection system is successfully adopted to complement *Bbtpx-1* in a *B*. *bovis Bbtpx-1* KO clonal line.

## Materials and Methods

### Parasites

The GFP-expressing and *Bbtpx-1* (BBOV_II004970) KO *B*. *bovis* lines were previously generated from the Texas strain of *B*. *bovis* [16]. The parasites were maintained with purified bovine red blood cells (RBC, Nippon Bio-Supply Center, Tokyo, Japan) in GIT medium (Wako Pure Chemical Industries, Osaka, Japan) by a microaerophilic stationary-phase culture system [17]. WR99210 (Jacobus Pharmaceuticals) was added to the culture media at 10 nM in order to maintain the GFP expression plasmids in the parasite.

### Transfection constructs

**Fig 1:**
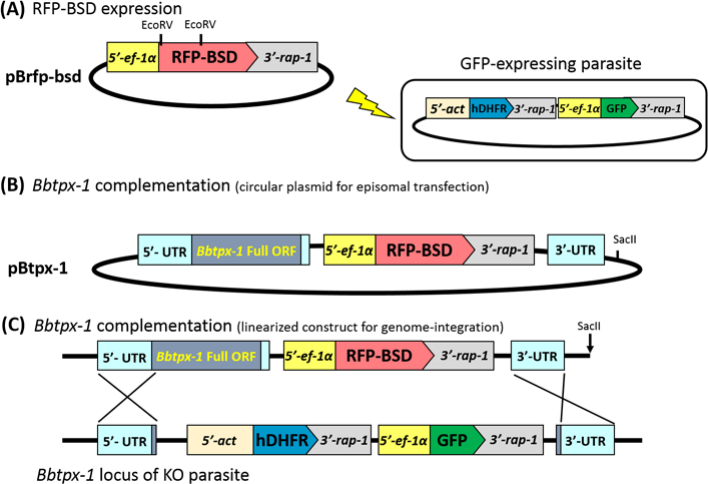
Schematic of the plasmid constructs used for transfection of *B*. *bovis*. (A) Restriction sites and expression cassette components of a plasmid pBrfp-bsd. *5'-ef-1α*, 5'-flanking region of the elongation factor-1α gene; *3'-rap-1*, 3'-flanking region of the rhoptry associated protein-1 gene; *rfp-bsd*, gene encoding a fusion protein consisting of red fluorescent protein and blasticidin-S deaminase. The plasmid already maintained in the GFP-expressing *B*. *bovis* parasite is also shown. (B) To complement *Bbtpx-1* expression, the full ORF of *Bbtpx-1* with its 5' and 3' UTR as a *Bbtpx-1* expression cassette, and *Bbtpx-1* 3' UTR region as a homologous sequence were cloned into pBrfp-bsd plasmid. (C) SacII site was used to linearize the plasmid for genome integration purpose. Abbreviations: 5'-*act*, 5'-flanking region of the actin gene; *dhfr*, human dihydrofolate reductase gene; and *gfp*, green fluorescent protein gene.

The pBrfp-bsd plasmid (Fig 1A) was designed to express a RFP-BSD fusion protein under the *ef-1α* promoter. The *rfp* and *bsd* genes were amplified by PCR from the monomeric *rfp* expression plasmid (kindly provided by Dr. Masatani, Kagoshima University) using the primer pairs 5’-AATTGATATCATGGCCTCCTCCGAGGAC-3’ and 5’-AATTGATATCGGCGCCGGTGGAGTGGCG-3’ (EcoRV sites are underlined), and the *gfp-bsd* expression plasmid [12] using primer pairs 5’-CGCCGATATCGAATTCCAGGCCAAGCCTTTGTCT-3’ and 5’-CATCCTGCAGGAATTTTATAAACGCATCTCATC-3’ (In-Fusion HD cloning sites are underlined), respectively. The amplified *rfp* gene was cloned into the EcoRV site of a plasmid containing the 5'-flanking region of the *ef-1α* gene (*5'-ef-1α*) and the 3'-flanking region of the *rap-1* gene (*3'-rap-1*) [12, 16]; followed by insertion of the *bsd* gene into the EcoRI site by In-Fusion system (Takara Bio Inc., Otsu, Japan). To construct the *Bbtpx-1* expression plasmid pBtpx-1 (Fig 1B and 1C), the *Bbtpx-1* gene locus (733 base of 5' untranslated region [UTR], *Bbtpx-1* full open reading frame [ORF] region and 66 base of the 3' UTR) and 3'-portion of *Bbtpx-1* were independently amplified by PCR from *B*. *bovis* genomic DNA (gDNA) and inserted upstream and downstream of the RFP-BSD expression cassette in the XhoI and NotI site of the pBrfp-bsd plasmid, respectively. The *Bbtpx-1* gene locus was amplified with the primers, 5’- CGGGCCCCCCCTCGAATTGCAAGGCAACCAATTTAC -3’ and 5’- TACCGTCGACCTCGATCGCATTTCGCATTAATTCAC -3’ (In-Fusion HD cloning sites are underlined). The 3'-portion of *Bbtpx-1* was amplified with the primers, 5’- AGTTCTAGAGCGGCCTTCGTTAATGCCTTCAACTCG-3’ and 5’- ACCGCGGTGGCGGCCGAATCTGCAGCAACCATTAGC -3’ (In-Fusion cloning sites are underlined). The primers were designed based on genome sequence information available in PiroplasmaDB (http://piroplasmadb.org/piro/). Constructed plasmids were purified using Qiagen Plasmid Midi Kit (Qiagen, MD, USA) following the manufacturer’s instructions and inserted DNA sequences were confirmed by sequencing.

### Transfection of *B*. *bovis*

*B*. *bovis* was transfected using a Nucleofector device (Amaxa Biosystems, Cologne, Germany) as described [16]. Briefly, 100 μl of infected RBC (IRBC) at 5–8% parasitemia were transfected with 10 μg of plasmid DNA in 100 μl of AMAXA nucleofector human T-cell solution (Amaxa Biosystems). To promote genome integration, the plasmid construct was linearized before transfection by overnight incubation with SacII. The plasmid-IRBC mixture was subjected to an electric pulse from the v-024 program of the Nucleofector device and was immediately transferred into 1 ml of culture medium containing 10% bovine RBCs. Blasticidin-S (Invitrogen, CA, USA) was added to the culture medium 24 hours after the transfection at a final concentration of 4 μg/ml. Parasites with RFP fluorescence signal were typically observed approximately 10 days after transfection. Following drug selection, parasites transfected with the linearized construct were cloned by limiting dilution by seeding approximately 0.5 IRBC per well in a 96 well plate. Several clonal parasite lines were obtained after 2 weeks of cultivation.

Successful transfections were assessed by diagnostic PCR assays, sequencing of PCR products, and Southern blotting. IRBC were lysed with PBS containing 0.15% saponin (Wako) and DNA was extracted using a QIAamp DNA Mini Kit (Qiagen, Hilden, Germany). PCR amplifications were performed using the primers Bbtpx-1 5’ F (5’-ACGCAGCATTCAGTTACTTCG-3’), Bbtpx-1 R (5’-AACTTCACCGTGCTTCTCGT-3’), dhfr F (5’-ATGGTTGGTTCGCTAAACTGC-3’), bsd F (5’-CAGGCCAAGCCTTTGTCT-3’), and Bbtpx-1 3’ R (5’-GTCATCGTTGGGTTTGACCT-3’); followed by sequence determination of PCR products. PCR reaction mixture was prepared according to the manufacture’s instruction of Tks Gflex DNA polymerase (Takara Bio). PCR condition was; one cycle of pre-denaturation at 94°C for 1 min, 35 cycles of denaturation at 94°C for 15 sec, annealing at 55°C for 20 sec, and elongation at 68°C for 1min (for PCR with Bbtpx-1 5’ F and Bbtpx-1 R) or 4 min (for PCR with dhfr F or bsd F and Bbtpx-1 3’ R), and one cycle of post-elongation at 68°C for 3 min. DNA sequences of PCR products were determined by primer-walking. For Southern blot analysis, 2.5 μg of the parasite gDNA was digested with XbaI, separated via agarose gel electrophoresis, and transferred onto a nylon membrane (Hybond N+; GE healthcare, Buckinghamshire, UK). The 5’*-Bbtpx-1* probe was prepared by PCR amplification using primers 5’-AATTCTCGAGATTGCAAGGCAACCAATTTAC-3’ and 5’-AATTCTCGAGTCGCATTTCGCATTAATTCAC-3’. Hybridization and detection was carried out using AlkPhos Direct Labelling Reagents and CDP-Star Detection Reagent (GE healthcare).

### Analyses of mRNA and protein expression

Parasite cultures were pelleted by centrifugation, supernatants discarded and total RNA extracted using the TRIzol reagent (Invitrogen) and purified using the SV Total RNA isolation system (Promega, WI, USA). Complementary DNA (cDNA) was prepared by RNase I treatment followed by reverse transcription using SuperScript III First Strand Synthesis System (Invitrogen). *Bbtpx-1* transcripts were detected by PCR from cDNA using primers as described [16]. As a control, cDNA of the methionyl-tRNA synthetase gene was detected using primers 5’-GGTACCGATGAGCATGGACT-3’ and 5’-GACGTGGCTCCGTAGTTCTC-3’. Indirect immunofluorescence assay was performed as described [18, 19]. Thin smears of IRBCs were fixed with 50% mixture of acetone/methanol and blocked with PBS containing 10% normal goat serum (Life technologies, NY, USA). Anti-BbTPx-1 rabbit serum was previously generated [16] and used at 1:200; and Alexa-Fluor 594 conjugated goat anti-rabbit IgG (Molecular probes, OR, USA) was used at 1:500. Immunofluorescence was detected using a laser scanning confocal microscope (Nikon A1R, Nikon, Tokyo, Japan) equipped with a × 60 objective lens (Nikon). For Western blot analysis, parasite pellets were prepared from IRBCs and solubilized by boiling in SDS-PAGE loading buffer (0.1 M Tris-HCl pH 6.8, 5% SDS, 15% glycerol, 4.5% dithiothreitol) containing 10% 2-mercaptoethanol. Protein concentrations were determined with a BCA protein assay kit (Thermo scientific) and 10 μg of the protein was applied in duplicate lanes for separation by 15% SDS-PAGE. One set of the duplicate lanes were stained with Coomassie Brilliant Blue (Rapid stain CBB kit; Nacalai tesque, Kyoto, Japan) and served as loading controls, and the other set was used for Western blot analysis. Parasite proteins were transferred to a polyvinylidene difluoride membrane (Clear Blot membrane-P; ATTO, Tokyo, Japan). Membranes were blocked with a blocking solution (Blocking One; Nacalai tesque) and reacted with anti-BbTPx-1 rabbit serum at 1:500. Afterward the membrane was washed with PBS containing 0.05% Tween 20, and the horseradish peroxidase (HRP)-conjugated goat anti-rabbit IgG (Promega W4018) was applied at 1:30,000. The signals were developed with Immobilon Western Chemiluminescent HRP Substrate (Millipore, MA, USA) and detected by LAS-4000 mini luminescent imaging analyzer (Fujifilm, Tokyo, Japan).

### Phenotypic analysis based on the sensitivity to nitrosative stress and oxidative stress

To evaluate the sensitivity of parasites against nitrosative stress or oxidative stress, parasites were maintained in the presence of a nitric oxide donor or super oxide donor, sodium nitroprusside (SNP, Sigma-Aldrich, MO, USA), at concentrations of 0, 1, 5, 10, 20 and 50 μM; or paraquot (Wako) at concentrations of 0, 0.1, 0.5, 1, 5 and 10 μM, respectively [20]. Stock solutions were prepared in DMSO such that final 0.5% DMSO was included in the culture medium. Cultures were initiated at a parasitemia of 0.1% in triplicated wells and culture medium was replaced every day. After four days, Giemsa-stained parasite smears were prepared and the number of parasites in 5,000 RBCs were counted and used to evaluate parasitemia. Statistical analysis was performed by a Student *t*-test and probability value of less than 5% (*p* < 0.05) was considered statistically significant.

## Results

### Establishment of sequential transfection method in *B*. *bovis* with WR99210 and blasticidin-S

**Fig 2:**
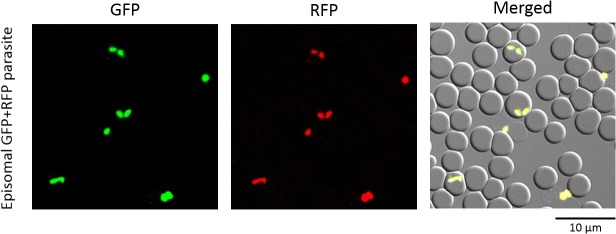
Live fluorescence images of *B*. *bovis* parasites. Parasites were episomally transfected with both RFP-BSD and GFP-hDHFR expression plasmids and selected with blasticidin-S and WR99210. Differential interference contrast and fluorescent images are merged on the right panel (Merged).

To develop a sequential transfection method in *B*. *bovis*, a plasmid expressing a RFP-BSD fusion protein (pBrfp-bsd, Fig 1A) was transfected into a previously established transfected parasite line expressing GFP from episomally maintained plasmids growing in *in vitro* cultures under WR99210 drug pressure [16]. After transfection and additional drug selection with 4 μg/ml blasticidin-S, RFP-positive parasites emerged as early as 10 days. Once RFP-expressing parasites were confirmed, transfected parasites were maintained with both 10 nM WR99210 and 4 μg/ml blasticidin-S. Both RFP and GFP fluorescence signals were consistently observed for more than three months under drug pressure (Fig 2). The transfectant parasite line showed comparable growth to the parental parasite line even when the culture medium was supplemented with both selection drugs (data not shown). Removal of the selection drugs resulted in the emergence of parasites expressing only RFP or GFP (data not shown), suggesting that under dual drug pressure both plasmids were episomally and independently maintained in the parasites and that both drugs were required to maintain the two episomal plasmids within a single cell. Since the sequential transfected parasites can be easily maintained under both blasticidin-S and WR99210 selection, these results indicate that WR99210/*hdhfr* and blasticidin-S/*bsd* selection systems can be used simultaneously in *B*. *bovis*.

### Generation of transgenic parasites that complement BbTPx-1 protein in the *Bbtpx-1* KO parasite line from episomal or genome-integration constructs

**Fig 3:**
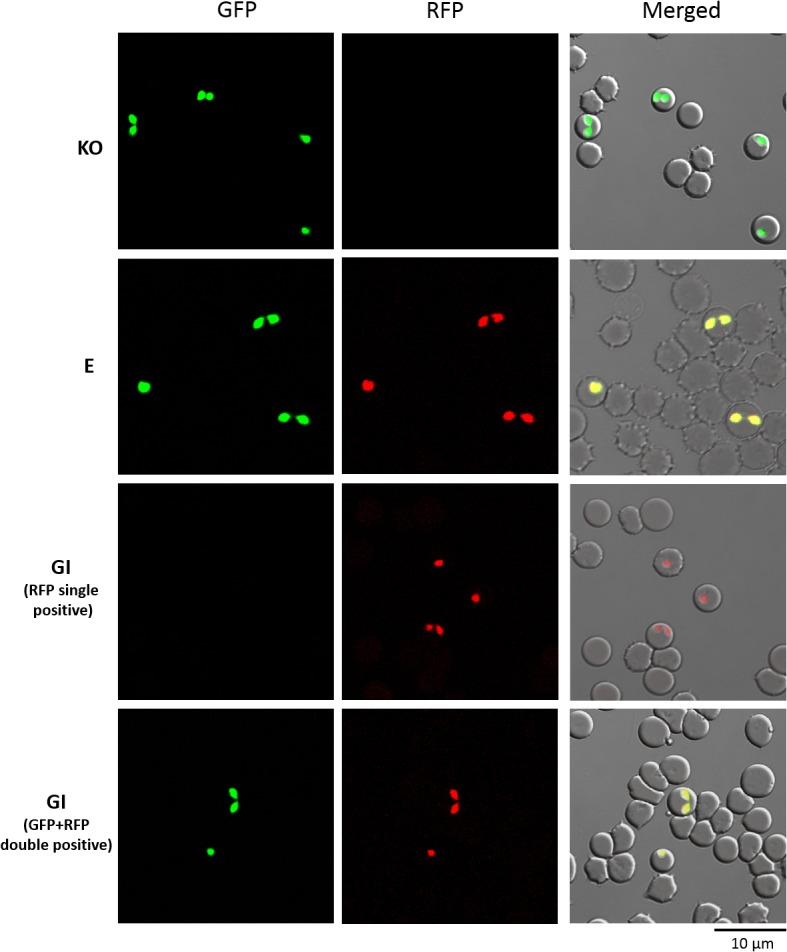
Live fluorescence images of *Bbtpx-1* KO parasites and *Bbtpx-1*-complemented transfectants. KO, *Bbtpx-1* KO parasites were previously derived (Asada et al., 2012). GI, cloned *Bbtpx-1* genome integrant. E, *Bbtpx-1* episomal transfectant. Differential interference contrast and fluorescent images are merged on the right panel (Merged).

Next, the double drug selection method was applied toward gene complementation in *B*. *bovis*. Parasites were transfected with a circular form of pBtpx-1 (Fig 1B), which was designed based on the RFP-BSD expression plasmid to introduce a functional BbTPx-1 expression cassette into the *Bbtpx-1* KO parasite line that expressed GFP and hDHFR from the disrupted *Bbtpx-1* gene locus (Fig 1C). Episomal transformants were successfully obtained under WR99210 and blasticidin-S double drug pressure, and all parasites were positive for both GFP and RFP fluorescence (Fig 3).

To attain genome integration of introduced DNA, we also transfected linearized versions of an expression plasmid construct (Fig 1C). After maintaining parasites under blasticidin-S pressure, two types of the parasite population emerged; one positive for only RFP fluorescence, and the other double positive for GFP and RFP fluorescence. After limiting dilution, clones were obtained positive only for the RFP signal, as well as clones double positive for both GFP and RFP (Fig 3). We interpret that in the former clones, the GFP-expression cassette that has been integrated to disrupt the *Bbtpx-1* gene locus was likely replaced with the introduced construct expressing RFP-BSD and BbTPx-1; whereas in the latter clones, the introduced construct was integrated into *Bbtpx-1* gene locus but in a manner not replacing the GFP-expressing cassette (result of diagnostic PCR is described in the next subsection). Parasite clones positive only for RFP fluorescence were selected for further expression and phenotype analyses.

### Confirmation of the linearized construct integration into the *Bbtpx-1* genome locus

**Fig 4:**
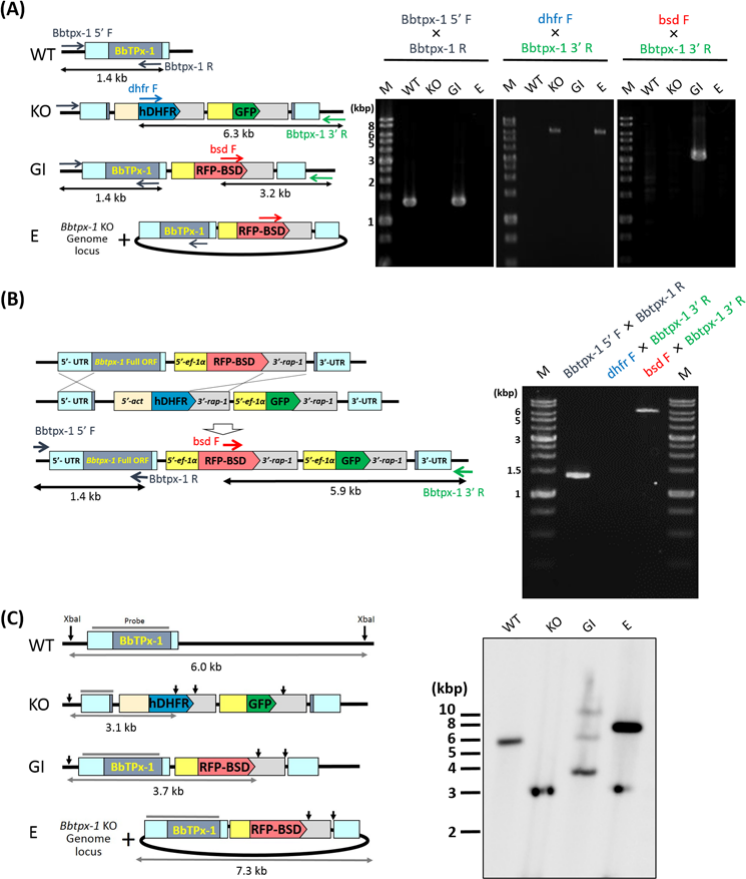
Analyses of the genome integration of transfected parasites. WT, wild type parental strain; KO, *Bbtpx-1* KO parasite; GI, *Bbtpx-1* genome integrant; and E, *Bbtpx-1* episomal transfectant. (A) Regions for *Bbtpx-1* (Bbtpx-1 5’ F×Bbtpx-1 R), hDHFR expression cassette (dhfr F×Bbtpx-1 3’ R), and RFP-BSD expression cassette (Bsd F×Bbtpx-1 3’ R) were PCR-amplified with specific sets of primers from parasites' gDNA. M, 1 kb DNA ladder marker. (B) Evaluation of a cloned genome integrant, which showed both GFP and RFP fluorescence. The proposed structure of the *Bbtpx-1* gene locus based on the diagnostic PCR assay results is shown in the left diagram. (C) Southern blot analysis. *Bbtpx-1* 5' UTR probe is indicated. Arrows in the schematic diagram indicate the expected location recognized by XbaI used for the digestion.

To confirm the integration of the linearized construct, firstly we performed PCR amplification and sequencing of the resulting amplicons. PCR amplification with primer sets hybridizing to the upstream of the expected 5' recombination site (Bbtpx-1 5' F) and *Bbtpx-1* ORF region (Bbtpx-1 R) yielded identical 1.4 kb PCR products from wild type parasite and the parasite clone transfected with the linearized construct (Fig 4A). In contrast, no amplicons were generated from *Bbtpx-1* KO parasites and the episomal transfectant using the same PCR primers with this PCR condition (Fig 4A). Consistently, PCR using primer sets hybridizing to *hdhfr* ORF region (dhfr F) and 3' downstream of the expected 3' recombination site (Bbtpx-1 3' R) amplified a 6.3 kb product from *Bbtpx-1* KO parasite and the episomal transfectant, but not from wild type parasites or the parasite clone transfected with the linearized construct. Moreover, PCR amplification with a primer for *bsd* ORF region (bsd F) and Bbtpx-1 3' R primer amplified a 3.2 kb product from the parasites transfected with the linearized construct, but not from the other parasites (Fig 4A). Nucleotide sequences of these amplified PCR products confirmed correct integration, and were deposited to DDBJ/EMBL/GenBank nucleotide database with the accession numbers LC006972 (1.4 kb PCR product), LC006974 (6.3 kb PCR product), and LC006973 (3.2 kb PCR product), respectively. These results support that the hDHFR and GFP expression cassettes in *Bbtpx-1* KO parasites are replaced by a RFP-BSD expression cassette and that the *Bbtpx-1* ORF region is precisely inserted into the original *Bbtpx-1* locus in the parasite clone transfected with the linearized construct.

In addition, we analyzed the genome of one cloned parasite line that expressed both GFP and RFP. PCR amplification with Bbtpx-1 5' F and Bbtpx-1 R primer sets yielded a 1.4 kb product and no amplicons were PCR-amplified with dhfr F and Bbtpx-1 3' R primers, indicating that *Bbtpx-1* ORF region was inserted into the original *Bbtpx-1* locus as expected (Fig 4B). On the other hand, PCR amplification with bsd F and Bbtpx-1 3' R primer sets yielded a 5.9 kb product from the parasite. These results were consistent with the expectation that *rap-1* 3' UTR sequence of RFP-BSD cassette was recombined with the *rap-1* 3' UTR sequence in the hDHFR cassette (Fig 4B left diagram).

To analyze the presence of episomal plasmid and the possible insertion of the linearized construct into genome region other than *Bbtpx-1* gene locus, Southern blot analysis was performed. Genomic DNA extracted from parasites were digested with XbaI and probed with the *Bbtpx-1* 5' UTR + ORF sequence. A single 6 kb or 3.1 kb band was detected from wild type or KO parasites, respectively, as expected (Fig 4C). This probe detected a strong 3.7 kb band, as expected, and weaker bands at ~6 and >10 kb from the parasite clone transfected with the linearized construct. These weaker bands suggest that the construct might also be integrated into additional genome sites. Nonetheless, these results confirmed that the linearized construct was correctly inserted into the genome locus, and thus we herein designated this transfectant as a genome integrant. Analysis of the episomal transfectant detected a 3.1 kb band, expected from the parasite's genome, and a stronger 7.3 kb band expected from the plasmid. The band at 7.3 kb was not detected from the genome integrant. These results indicate that no episomal plasmids are remaining in the genome integrant, and that genome integration of the construct did not occur in the episomal transfectant.

### Validation of the BbTPx-1 expression in the transfectants

**Fig 5:**
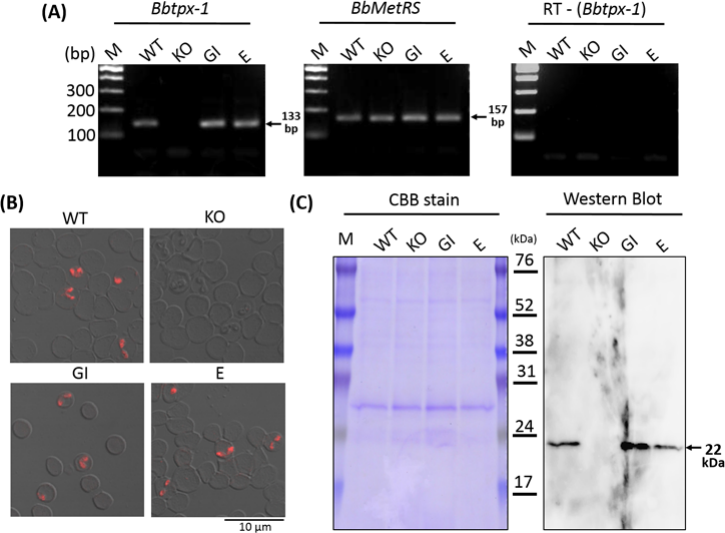
Validation of the BbTPx-1 expression in the transfectants. Expression of *Bbtpx-1* in the wild type parental strain (WT), *Bbtpx-1* KO parasites (KO), *Bbtpx-1* genome integrant (GI), and *Bbtpx-1* episomal transfectant (E). (A) RT-PCR assays were performed for *Bbtpx-1* gene, methionyl-tRNA synthetase gene (BbMetRS), and a negative control without reverse transcription (RT—(*Bbtpx-1*)). M, 100 bp DNA ladder marker. Expected RT-PCR band size of 133 bp and 157 bp are indicated for *Bbtpx-1* and *BbMetRS*, respectively. (B) Indirect immunofluorescence assay with anti-BbTPx-1 serum. Differential interference contrast and fluorescence images are merged. The red fluorescence in the cytoplasm of the complemented transfectants indicates expression of BbTPx-1. (C) Western blot analysis with anti-BbTPx-1 serum. A band was detected from WT, GI, and E, which is consistent to the expected size of BbTPx-1 protein of 22 kDa. The CBB staining image in the left panel shows equal loading.

Restored expression of BbTPx-1 in the transfectants was evaluated at the transcription and protein expression levels. RT-PCR amplification with specific primers for the *Bbtpx-1* gene demonstrated a positive 133-bp band only from wild type parasites and *Bbtpx-1* complemented transfectants, but not from the KO parasite (Fig 5A left panel). A 157-bp product was amplified with the primers targeting methionyl-tRNA synthetase gene from all samples, demonstrating successful extraction of the cDNA; and no amplification from the RT negative control, discounting the possibility of gDNA contaminating the RNA extracts used in the RT-PCR reactions (Fig 5A middle and right panels). Indirect immunofluorescence assay with anti-BbTPx-1 serum revealed red fluorescence inside the wild type parasite, episomal transfectant, and genome integrant; suggesting a cytoplasmic localization of BbTPx-1 protein (Fig 5B). Western blot analysis with anti-BbTPx-1 serum detected 22 kDa band in wild type, episomal transfectant, and genome integrant, confirming BbTPx-1 protein was expressed in these parasites (Fig 5C). Consistently, anti-Bbtpx-1 serum did not detect signals in *Bbtpx-1* KO parasites by either IFA or Western blotting (Fig 5B and 5C), indicating no BbTPx-1 protein expression in the KO parasites. These results indicate that BbTPx-1 protein was successfully expressed from the BbTPx-1 expression cassette maintained episomally or integrated in genome in the *Bbtpx-1* KO parasite.

### Parasite resistant property to nitrosative stress is reduced in *Bbtpx-1* KO parasite, which can be restored by complementation of *Bbtpx-1*

**Fig 6:**
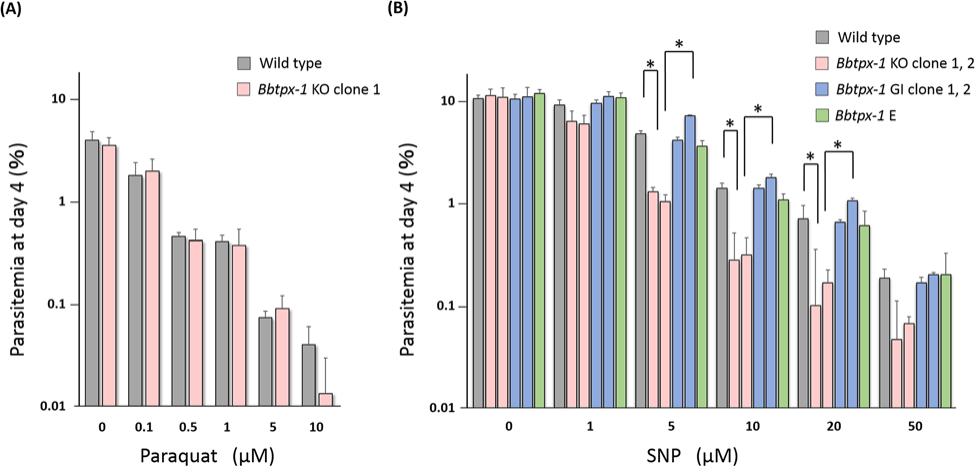
Sensitivity of the parental wild type (WT) parasites, *Bbtpx-1* KO (KO), *Bbtpx-1* genome integrant (GI), and *Bbtpx-1* episomal transfectant (E) to paraquat and SNP. (A) Sensitivity of the *Bbtpx-1* KO parasites to paraquat. *Bbtpx-1* KO parasites and parental wild type parasites were cultured in the medium with 0, 0.1, 0.5, 1, 5 and 10 μM paraquat. The cultures were initiated with 0.1% parasitemia and parasitemias at day 4 were plotted. (B) Sensitivity of the *Bbtpx-1* KO and *Bbtpx-1* complemented parasites to SNP. The parasites were cultured in the medium with 0, 1, 5, 10, 20 and 50 μM SNP. Two clones for *Bbtpx-1* KO parasites and genome-integrated *Bbtpx-1*-complemented transfectants were used in this experiment. The parasitemia is mean ± S.D. of the triplicated well culture (*: p < 0.05 by Student *t*-test).

In our previous study we did not extensively analyze phenotypic changes in the *Bbtpx-1* KO parasite line [16], and therefore herein we evaluated whether disruption of BbTPx-1 alters resistance to oxidative and nitrosative stresses. We found that 4 days exposure to paraquat, a superoxide donor, did not show any difference in the effect with respect to the wildtype line (Fig 6A). However, after 4 days exposure to SNP, a nitric oxide donor, the parasitemia of the *Bbtpx-1* KO parasite was significantly lower than wild type parasites at concentrations of 5, 10 or 20 μM (*p* < 0.05, Fig 6B). Consequently, phenotypic complementation of the *Bbtpx-1*-complemented transfectants was assessed by *in vitro* cultivation with SNP. The parasitemia of two clones of *Bbtpx-1* genome integrant and episomal transfectant after 4 days exposure to SNP was significantly higher than that of *Bbtpx-1* KO parasite (*p* < 0.05), and comparable to that of wild type parasite at concentrations of 5, 10 and 20 μM (Fig 6B). These results indicate that the reduced resistance to nitrosative stress of the *Bbtpx-1* KO parasites was restored in both types of *Bbtpx-1*-complemented transfectants.

## Discussion

In this study we complemented for the first time a previously disrupted gene in *B*. *bovis*, by using blasticidin-S/*bsd* selection system as a second selectable marker. The number of available drug selection markers is one limiting factor impeding the development of gene disruption/complementation experiments aimed at assessing gene function in cells. In *B*. *bovis*, thus far two distinct types of transgenic *B*. *bovis* parasite lines have been produced to express GFP: [i] a blasticidin-S resistant genome integrant line expressing GFP-BSD fusion protein from the *Bbef-1α* locus of the parasite [12]; and [ii], a WR99210 resistant episomal transfectant expressing GFP using *hdhfr* as a selectable marker [16]. In this study, we combined these two selectable markers within a gene complementation experiment to analyze gene function in *B*. *bovis*.

Linearized pBbtpx-1 was designed to replace the GFP-hDHFR expression cassette with RFP-BSD, and BbTPx-1 expression cassettes by double crossover at the 5’ and 3’ UTRs of *Bbtpx-1* gene locus. However, nearly half of the parasites obtained upon transfection showed both RFP and GFP fluorescence. Using PCR primers diagnostic of integration into the *Bbtpx-1* ORF region and the RFP-BSD cassette, which observed apparent recombination both within the *Bbtpx-1*, as anticipated, and also at the 3' site within the *rap-1* 3' UTR in the hDHFR cassette. These results are indicative of potential undesirable integration problems due to repetitious DNA sequences (in our case, *rap-1* 3' UTR and *ef-1α* 5' UTR) present during sequential or simultaneous transfections.

Furthermore, in the RFP positive and GFP negative parasites, the results of diagnostic PCR and Southern blot analysis were consistent to the expectation that the construct was inserted into the *Bbtpx-1* locus, however the Southern blot probe detected additional weak bands only in the genome integrant parasites. Similar phenomenon was also observed in the first report which the GFP-BSD expression construct was inserted into the *ef-1α* locus [12]. Until now, we have not observed the genome integration of the plasmid by non-homologous recombination in *B*. *bovis* in our hands. In addition, inter- and intrachromosomal segmental gene conversion were reported among *ves* multigene family members in *B*. *bovis* [21], so it is possible that partial sequence of *Bbtpx-1* was integrated or converted into the genome as a result of transfection. To date, a limited number of promoter and terminator sequences have been available for *B*. *bovis* transfection [12, 15]. Therefore, further promoter and terminator regions need to be identified in order to develop more efficient and precisely targeted genome-integrated sequential transfection system. Because BbTPx-1 was successfully expressed in the episomal transfectant, 5' and 3' UTRs of the BbTPx-1 expression cassette in this plasmid can be used in future studies. In addition, the 5’ and 3’ UTRs validated in the related apicomplexan parasites, such as *Plasmodium* spp. and *Toxoplasma gondii*, may also be tested, which would provide advantages to avoid problems of undesirable integration.

Thioredoxin peroxidases are a ubiquitous family of antioxidant enzymes [22]. In published studies we found disruption of the *tpx-1* gene in either *B*. *bovis* or *P*. *falciparum* did not affect their growth under normal *in vitro* culture conditions [16, 20]. In the case of *P*. *falciparum*, the growth of *tpx-1* KO line was suppressed in the presence of paraquat, an oxidative stress, or the nitrosative stress, SNP, in comparison with wild type parasites [20]. In this study, however, we show a role of *B*. *bovis* TPx-1 in resistance to nitrosative stress, but not to the oxidative stress caused by paraquat at concentrations up to 10 μM. This discrepancy between *B*. *bovis* and *P*. *falciparum* in the response of the *tpx-1* KO line to oxidative stress may be explained by a presence of a functional compensation from other peroxide-reducing enzymes, such as catalases in *B*. *bovis*. For example, enzymatic activity of catalase has been reported in *Babesia* spp., but not in *Plasmodium* spp. [23].

The double selection techniques described herein provide a platform to generate double gene knockouts in *B*. *bovis*. Double gene knockout approaches will accelerate the functional study of molecules encoded by multigene families and may allow future development of live attenuated vaccines upon the deletion of multiple virulence factor genes. Nonetheless, additional transfection methods need to be developed for further investigating the biology of *Babesia* parasites. In recent years, several transfection methodologies such as FLP/FRT-mediated conditional mutagenesis, a tetracycline-repressible transactivator system, and CRISPR/Cas9 genome editing system have been successfully used for the other apicomplexan parasites [24–26]. The combination of blasticidin-S/*bsd* and WR99210/*hdhfr* selection systems developed in this study is ready to adapt to new techniques in transfection-based research on the biology of *Babesia* parasites.
